# Non-parametric spatiotemporal trends in fire: An approach to identify fire regimes variations and predict seasonal effects of fire in Iran

**DOI:** 10.1371/journal.pone.0319993

**Published:** 2025-04-04

**Authors:** Peyman Karami, Sajad Tavakoli

**Affiliations:** 1 Department of Environmental Sciences, Faculty of Natural Resources and Environment Sciences, Malayer University, Malayer, Iran; 2 Department of Environmental and Forest Science, Faculty of Natural Resources and Environment, Science and Research Branch, Islamic Azad University, Tehran, Iran; Bowling Green State University, UNITED STATES OF AMERICA

## Abstract

Analyzing wildfire complexity provides valuable insights into fire regimes and occurrence patterns within landscapes, enabling targeted land management efforts for sensitive and vulnerable areas. Fire density is a key component of wildfire regimes. In recent years, Iran has experienced significant changes in wildfire activity. This study aims to assess trends in fire density and the probability of wildfire occurrence during summer and autumn using active fire data. Seasonal fire point density (per km^2^) from 2001 to 2023 was calculated using a kernel function. The Mann-Kendall (MK) test identified areas with significant fire density trends (at the 90% confidence level) for prediction analysis. Environmental variables and points with significant trends were entered into a MaxEnt model to predict fire risk in summer and autumn. Environmental variables included average temperature, human modification of terrestrial systems, annual precipitation, precipitation of the driest month, elevation, land use/land cover (LULC), land surface temperature (LST), soil organic carbon (SOC), and wind exposure index (WEI). Spatial variations in significant fire density trends for summer and autumn were analyzed using gap analysis and the Kappa index. Influence zone analysis identified zones impacted by increasing wildfire trends within the landscape. Results showed that areas with significant increasing fire density trends covered 326,739.56 km^2^ in summer and 102,668.85 km^2^ in autumn. There was minimal overlap between increasing and decreasing fire density trends across seasons, indicating wildfires disproportionately affect natural and agricultural areas in Iran. Influence zone analysis identified 15 fire-prone zones in summer and 3 in autumn, with a significant portion located in the Zagros Mountain forest steppes. The MaxEnt model, based on the area under the curve (AUC) metric, successfully identified high-risk wildfire areas in both seasons. Jackknife analysis indicated that human modification and SOC were crucial indicators of human activities and available fuel for wildfires in both seasons. Predictions showed diverging wildfire risk patterns in summer and autumn. In summer, wildfire risk is high across all regions except deserts and Hyrcanian forests, while in autumn, Hyrcanian mixed forests are also classified as high-risk zones. These findings can help land managers identify influence zones and understand the land uses and vegetation types associated with wildfires, enabling more informed and effective management decisions based on the spatial extent and distribution of fire trends.

## 1. Introduction

The Middle East is grappling with numerous environmental crises, including climate change, dust storms, and wildfires, which have become increasingly apparent in recent years due to rising temperatures. Wildfires, defined as uncontrolled fires affecting vegetation, forests, and other natural landscapes, have far-reaching impacts on the environment, economy, and society [1]. Fire is a complex phenomenon shaped by physical, chemical, and environmental factors. Extreme fire weather conditions are expected to occur over longer fire seasons in many regions [2]. Fires contribute to air pollution, soil erosion, and water contamination, with long-term effects on ecosystems and human health [3]. Each year, fires of varying intensity affect forests worldwide [4]. According to Andela et al. (2019) [5], between 2003 and 2016, approximately 3.13 million fires occurred globally, each covering an area of at least 21 hectares. In the Middle East, wildfires have become a serious concern due to their devastating impacts on human lives and the environment [6]. Wildfires are common across Iran’s forests and rangelands, leaving these ecosystems highly vulnerable [7,8]. Furthermore, fires can undermine the long-term resilience of old-growth forests [9]. A study by Heydari et al. (2017) [10] on the effects of fire severity on soil in the Zagros oak forests of Iran revealed that high-severity fires increased soil acidity, electrical conductivity, and bulk density, while decreasing organic carbon, moisture content, and microbial activity. Fire also impacts the species inhabiting these ecosystems. For example, research has shown that in some protected areas in western Iran, particularly the Zagros forests, the increased frequency of fires [11] is affecting wildlife, with smaller species being most impacted [12].

Given the intricate nature of fire behavior, understanding past and current fire regimes is crucial for assessing the temporal and spatial patterns of fires [13]. Fire regimes describe the long-term average fire characteristics in a specific area, encompassing frequency, density, intensity, severity, and seasonality [14]. These regimes offer a framework for understanding fire behavior, its ecological role, and management strategies. For example, areas with dense vegetation and proximity to human settlements are more prone to frequent wildfires due to increased ignition sources and fuel availability [15]. Fire regimes are influenced by factors such as climate, land use, and topography [16].

Knowledge of fire regimes can assist in wildfire risk zoning and the identification of Influence Zones. Wildfire risk zoning involves the classification of Landscape into different certain wildfire risk zones based on the temporal patterns and changes in fire density over time. By analyzing the timing and frequency of historical fire occurrences, alongside relevant environmental factors, wildfire risk zones are identified. This approach allows for a dynamic understanding of wildfire risks, ensuring that the zones reflect the most current trends and patterns. Additionally, increasing the density of known wildfire samples and improving the spatiotemporal resolution of environmental data are crucial for the reliability of wildfire risk zones [17]. As a part of wildfire risk assessment, zoning studies provide an important scientific basis for building wildfire risk defense systems, deploying fire prevention forces, and guiding wildfire prevention work [17]. So, having an up-to-date map of wildfire risk zoning is of great of importance for conserving forest and rangelands [18].

Timely monitoring of ecosystem disturbances is crucial for rapidly evaluating and responding to their impacts on carbon dynamics, biodiversity, and socio-ecological systems [19]. Satellite remote sensing (SRS) has historically played a prominent role in observing Earth surface phenomena at various spatial and temporal scales. Analyzing the temporal dynamics of ecosystem components often involves using time-series data, a powerful method for addressing ecological questions [20,21]. Terrestrial ecosystems are dynamic, exhibiting both gradual and abrupt changes, and rarely remain static over time [22]. When Earth surface features are monitored by SRS at specific time scales, a time series of that feature can be formed. The goal of time-series analysis is to describe and quantify underlying dynamic behavior, link different observations, and uncover potential causes of observed phenomena [23]. SRS time series are becoming increasingly accessible due to new space missions providing high spatial resolution coverage of the Earth every few days [24]. The ability to select time scales allows SRS time series to be chosen at various intervals, such as annually [25], seasonally [26,27], and for animal species during breeding seasons [28]. Monitoring the dynamics of time-specific environmental indicators can simplify the analysis of environmental changes [28]. One such satellite system currently in use is Moderate Resolution Imaging Spectroradiometer (MODIS). The MODIS sensor, with its high radiometric resolution (12-bit), is carried by two U.S. satellites, Terra (operational since 2000) and Aqua (since 2002) [29]. These satellites can capture up to four images daily of areas experiencing wildfires [30]. In addition to tracking fires, SRS monitoring enables the observation of environmental variables that influence fire occurrence. Land surface temperature (LST) is among the most important SRS products, offering a precise measure of the Earth’s surface energy balance. By analyzing trends in LST alongside fire trends, the effects of climate change on wildfire patterns can be better understood.

The inherent unpredictability of fire, influenced by a myriad of environmental and climatic factors, poses significant challenges for effective fire management and mitigation [11]. To address this issue, machine learning (ML) methods have emerged as powerful tools for predicting fire occurrences by identifying complex patterns within large datasets [31]. The use of these techniques in fire modeling has demonstrated considerable potential in enhancing prediction accuracy and supporting decision-making processes [32]. Research by Alkhatib et al. (2023) [33] and Pham et al. (2020) [32] underscores the effectiveness of various ML approaches in forecasting and mapping fire susceptibility. Techniques such as Bayesian networks, decision trees, and multivariate logistic regression have been assessed for their ability to analyze fire behavior and forecast future events [32]. Furthermore, Bot and Borges (2022) [34] provide an in-depth analysis of how ML can aid wildfire management across multiple stages.

To establish a valid ML model, an adequate and reliable classified dataset is essential [35]. An accurate training dataset is critical, but assigning raw data to the appropriate class can be prone to errors. Detecting and eliminating outliers is crucial in nearly any quantitative field, including ML [36]. Various outlier detection methods, such as distance-based, density-based, clustering-based, and angle-based approaches, have been developed [37]. Filtering data based on significant trends in fire density, identified through time series processing, can enhance the predictive accuracy of ML models. Using historical fire data helps these models explain temporal trends and anomalies, improving their ability to predict future fire events with greater precision. This approach highlights the importance of data preprocessing in maximizing the effectiveness of ML algorithms in fire prediction and management. By analyzing wildfire time-series data, fire risk zoning can be established, informing spatially-prioritized management actions. Assessing fire risks timely and accurately, planning fire risk areas rationally, and allocating firefighting resources scientifically have become essential strategies for mitigating the threats of forest fires caused by climate change [38]. Such zoning facilitates the effective allocation of resources for fire prevention and suppression, including efficient monitoring of high-risk zones [39]. Non-parametric tests offer robustness and flexibility for time series analysis, especially when dealing with non-linear patterns or irregular data [40].

Wildfire occurrence is influenced by complex interactions among climate, fuel type, topography, vegetation, and human activities [41,42], all of which must be considered in the spatio-temporal analysis of fire. In addition to temperature and its changes, land use/land cover (LULC) is also an important criterion for fire. Changes in LULC, such as clear-cutting forests for agricultural development, indiscriminate livestock grazing, and activities of nomadic herders, have significantly altered vegetation cover [31]. Moreover, strong dry winds due to severe weather conditions can advance cloud-to-ground lightning-induced wildfires [43]. Wildfires consume large amounts of above-ground, topsoil, and sometimes underground biomass, often reducing the entire soil organic layer to ash [44]. This complete combustion significantly affects the nutrient cycle and soil fertility, leading to long-term ecological impacts. Soil organic carbon (SOC) contributes to the overall fuel load in an ecosystem. Higher levels of SOC can increase the amount of combustible material available, potentially intensifying fires [45]. Moreover, SOC is crucial for post-fire soil recovery, influencing vegetation regrowth and soil stability. Increases in the number of heatwaves, droughts, and dry spells should also be considered, as these climatic extremes exacerbate fire risk by drying out vegetation and reducing soil moisture levels [46].

On a global scale, several studies have examined wildfire trends. Yang et al. (2023) [47] explored global fire activity and the spatiotemporal patterns of LULC from 2001 to 2023. Their study found that most burned areas were detected in early spring and summer, with nighttime fire occurrences peaking in July. Burned areas in forests, grasslands, and croplands exhibited dual peaks in April and from July to September, while shrublands, barren lands, and wetlands saw peak fire activity in July or August. Earl and Simmonds (2018) [48] used MOD14A1 data to analyze global fire activity, indicating a decline in active fires in the Northern Hemisphere and North Africa between 2001 and 2016, largely due to agricultural expansion, while fire activity increased in China, India, and Southern Africa. Several studies have explored wildfire occurrences in Iran [16,49,50], but only a few have examined the trends and temporal dynamics of these events. Beygi Heidarlou et al. (2024) [51] investigated the role of climate and environmental dynamics in shaping fire patterns in the northern Zagros region. Their analysis examined annual fluctuations in fire activity using variables such as temperature, humidity, precipitation, wind speed, heat waves, and solar radiation, alongside multivariate linear regression to characterize fire behavior. The results emphasized the urgent need for intervention in the northern Zagros region. Similarly, Sayahnia et al. (2024) [12] assessed conservation priorities for mammals in response to fire in Zagros oak forests. This study analyzed fire data from 2000 to 2021 using time-scan statistical permutation and overlaid the results with a species richness map for 88 vertebrate species. Findings revealed an average of 76.2 fires per year in these forests, with the northwest Zagros showing the highest frequency and clustering of fires, highlighting the extensive spatial distribution of wildfire activity.

In Iran, most wildfire studies have focused on specific regions, provinces, or natural ecosystems, such as forests. Despite the abundance of research, there has been limited focus on fire density and the integration of spatial trends. Incorporating fire density data with spatial and temporal patterns could yield important insights into fire regimes. Through active fire and burned area products, SRS offers valuable tools for such analyses. Therefore, the goal of this study is to use MODIS active fire data to examine (1) trends in fire density, (2) Influence zones of fire, and (3) the factors influencing fire occurrence in Iran.

## 2. Materials and methods

### 2.1. Study area and fire points

**Fig 1:**
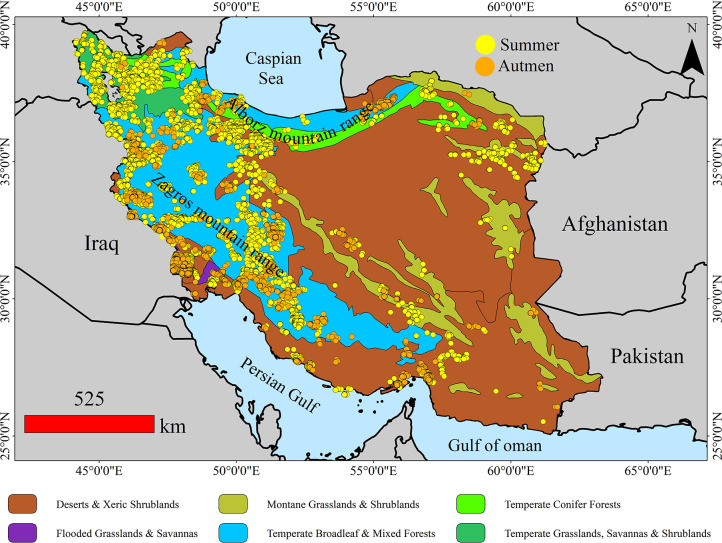
Samples within the range of increasing fire density trends, separated by summer and autumn, in different biomes of Iran.

Iran is located in West Asia, bordered by the Caspian Sea to the north and the Persian Gulf and Gulf of Oman to the south (Fig 1). The country’s landscape is predominantly mountainous, featuring major ranges such as the Zagros Mountains in the west and the Alborz Mountains in the north. These mountain ranges act as natural barriers, influencing both the climate and settlement patterns across the country. Fig 1 illustrates the geographical location of Iran and its ecoregions. The ecoregion map of Iran was obtained from https://ecoregions.appspot.com/.

In this study, wildfire activity was assessed using active fire data from the MODIS satellite (sourced from https://www.earthdata.nasa.gov/learn/find-data/near-real-time/firms). For the analysis, we utilized the MODIS MCD14DL Collection 6.1 active fire product, which is produced using a contextual algorithm that identifies fires occurring within 1-kilometer pixels that are actively burning at the time of satellite overpass, under relatively cloud-free conditions. This algorithm processes the MODIS data to detect fires, providing information on the time and geographic coordinates of fire events with high accuracy and minimal commission error (1.2%) [52]. Detection confidence of MODIS ranges from 0 to 100%, and above 30% is considered to have better accuracy [53]. 2. Therefore, we only included fire points with confidence levels exceeding 30% to ensure robust fire pattern analysis [54]. This threshold has been widely applied in other studies [55]. Each fire detection is represented as a point vector corresponding to the centroid of a 1 km ×  1 km pixel where one or more fires were detected during satellite overpass [53]. After collecting fire data from 2001 to 2023 and sorting the records by season, the Mann-Kendall (MK) test identified records with significant increasing trends in fire density. Points within the range of these increasing trends were then entered into the predictive analysis, resulting in 55,178 points for summer and 29,656 points for autumn.shows the location of these points by season.

### 2.2. Fire regime

To model wildfire risk, fire regimes were analyzed first. After isolating fire points with more than 30% confidence, wildfire density for 2001-2023 was calculated in ArcGIS 10.4.1 using the kernel density function [39]. A crucial component of this analysis is the search radius, also known as bandwidth. Since the sample size is larger during summer (June 21 to September 22) and autumn (September 23 to December 31), corresponding to more informative datasets, a finer cell size resolution is preferred [39]. In this study, cell size was set to 1000 meters, equal to the spatial resolution of the input data. For the MK test, all density maps were converted into ASCII format and imported into TerrSET 2020. The data were then organized into a raster group file and subsequently converted to a time series file in the Collection Editor. The Collection Editor, like TerrSet Explorer, provides the facility to create and edit raster group files, vector link files, signature group files, and hyperspectral signature group files.

Fire regime analysis was conducted using the non-parametric MK test, which has been used in past studies to analyze trends in LST [26–28,56], vegetation cover [56], inflow changes in wetlands [57], and habitat suitability under climate change scenarios [58]. The test generates several outputs, including a z-score, often referred to as the z-score of the MK test (ZMK) [56]. This metric measures the statistical significance of changes, with high values indicating increasing trends and low values indicating decreasing trends [28]. The MK test requires time series data to be serially independent, making autocorrelation in inputs a concern. The presence of serial correlation can make trend tests overly sensitive, leading to frequent rejection of the null hypothesis, especially with positive serial correlation [59]. Pre-whitening is a process used to remove serial correlation from time series data before applying the MK test. However, since this study utilizes a large dataset, the impact of autocorrelation is minimized, and there was no need for pre-whitening [27]. The variation and range of ZMK are discussed in Kandya et al. (2021) [60]. This study examined both increasing and decreasing wildfire density trends at significance levels above 90% (ZMK ˃  1.654 and ˂  -1.654), including 90%, 95%, and 99%. After setting the significance threshold, a binary map was created, with a value of 1 indicating significant increasing or decreasing trends in fire density, and a value of 0 indicating no significant trends. The threshold was applied using the Raster Calculator tool in ArcMap.

### 2.3. Spatio-temporal changes in fire regime

After identifying patches with significant changes during summer and autumn, these changes were compared using GAP analysis. This analysis is interpreted through three Kappa indices: overall Kappa, location Kappa, and histogram Kappa. This method is based on a cell-by-cell comparison of maps, where each pair of cells is checked for equality. Overall Kappa measures the overall agreement between trends, ranging from -1 to + 1. High agreement results in values close to + 1, while negative values indicate no agreement. To better interpret the Kappa index, two other indices were used: histogram Kappa and location Kappa. Histogram Kappa reflects the similarity in quantity, referring to the number of cells that fall within the same category. Location Kappa indicates the spatial distribution and agreement of categories [61]. The overall Kappa index is derived by multiplying these two indices. This analysis was conducted using the Map Comparison Kit [62]. After applying the threshold (ZMK ˃  1.654 and ˂  -1.654), distinct increasing and decreasing trends were observed for each season. The location of these trending areas was examined using the CROSSTAB tool in TerrSet, which performs a cross-tabulation analysis that compares images containing categorical variables of two types.

### 2.4. Influence zones of fire

Key factors influencing the fire regime include fire occurrence density, burned rate, and median fire size [15]. In this study, distance was used to identify Influence zones. Distance can influence fires due to the spatial distribution of factors such as vegetation, topography, and human activities [15]. The Graphical User Interface for Describing Image Objects and their Shapes (GUIDOS, http://forest.jrc.ec.europa.eu/biodiversity/GUIDOS/) was used for landscape-level analysis of Influence zones. The distance-based analyses available in GuidosToolbox (GTB) further illustrate the influence zones of each object and determine the pairwise proximity between neighboring image objects [63]. Influence zones in GTB are defined by the outer equal distance delimiter lines (isodistances) that separate selected foreground objects. These zones are derived using a morphological watershed operator on the Euclidean distance map of the background area [63]. The process helps identify boundaries where local minima act as drainage points, with small objects excluded to avoid over-segmentation. For the Influence zones analysis in GTB v3.3, areas with an increasing trend (more than 90 percent) in fire density were converted to map values of 0 and 1. Areas with a value of 2 were assigned as foreground, areas with a value of 1 as background, and areas outside the study area received a value of 0. Influence zones are driven by three key parameters: minimum area, foreground buffer zone, and background buffer zone, allowing for detailed customization and accurate delineation of the spatial impact of landscape features. The values for these three parameters remained as default.

### 2.5. Modeling fire regimes

#### Environmental variables.

Various environmental factors influence wildfire occurrence, generally grouped into four categories: climatic, topographic, vegetation, and human activities [64,65]. This study evaluated variables impacting wildfire occurrence, with an emphasis on research by Trucchia et al. (2022) [66] and Sari (2023) [67] that focused on fire incidents. Elevation data, with a spatial resolution of 30 seconds, was accessed from [https://www.worldclim.org/]. Digital elevation model (DEM) can be used to derive several environmental metrics, including wind exposure. Wind is one of the primary factors influencing the spread and intensity of wildfires [68]. To assess the impact of wind, we used the wind exposition index (WEI), where values below 1 represent wind-shadowed areas, and values above 1 indicate areas exposed to wind [69]. Precipitation patterns can significantly impact wildfires. Annual precipitation (Bio12) and precipitation of the driest month (Bio14) were obtained from the CHELSA database. The long-term annual average air temperature was obtained from the Solargis global solar model via https://globalsolaratlas.info, representing average temperatures from 1994 to 2018. In a study by Sinha et al. (2023) [70], surface temperature was identified as a key variable for fire zone classification.

However, temperature alone does not fully capture fire regime dynamics. Therefore, in addition to average temperature maps, LST trends were also examined. LST data, covering the summers and autumns from 2003 to 2023 were retrieved using the MYD11A1 MODIS product via Google Earth Engine [71]. For each season in each year, a mean LST map was generated to address potential bias due to MODIS’s preference for cloudless days [72]. The date of receiving the mean LST was set to coincide with the date of the fire point filter. LST trends were analyzed using the non-parametric MK test and the resulting ZMK was included as a variable in modeling. Fuel availability plays a central role in wildfire risk [73]. Therefore, selecting an environmental variable that accurately represents biomass availability is essential. While NDVI is commonly used, its seasonal variability and inability to reflect soil conditions make it insufficient. Instead, this study utilized soil organic carbon (SOC) as a proxy for biomass and soil characteristics [74]. SOC data was obtained from http://54.229.242.119/GSOCmap with a spatial resolution of 30 seconds. The continuity of fuel load is strongly influenced by LULC patterns. The Copernicus Climate Change Service (C3S) provides intermediate climate data records for many essential climate variables, including LULC. Annual LULC maps with a spatial resolution of 300 meters were obtained from C3S for 2016-2019. The data revealed 24 LULC classes in Iran. An important variable in this study was the Human Modification of Terrestrial Systems (HMTS), a continuous 0-1 scale that reflects the proportion of landscape modified by human activity. This metric is based on 13 anthropogenic stressors, modeled using spatially explicit global data, with a median year of 2016. Higher values indicate a stronger human influence, while lower values suggest more pristine landscapes. This variable was sourced from https://earthdata.nasa.gov with a spatial resolution of 30 seconds. This metric was classified according to Salafsky et al. (2008) [75] based on the following parameters:

1- Urban and built up areas (Built up) 2- Agriculture (Cropland, pasture lands, and grazing) 3- Energy production and mining (Oil and gas production) 4- Transportation and service corridors (Electrical infrastructure roads, railways, power lines and towers) 5- Biological harvesting (Logging and wood harvesting) 6- Human intrusions (Human intrusion) 7- Natural system modifications (Reservoirs) and 8- Pollution (Air pollution). To refine the analysis, a correlation test was performed using Band Collection Statistics in ArcMap; environmental variables with | r | > 0.85 were excluded from further analysis. All included variables had a spatial resolution of ~  1 km2 and data was prepared in ArcMap. Table 1 shows the variables that affect the probability of fire occurrence.

**Table 1:** Environmental variables used in the analysis of wildfires in Iran (2001–2023).

Variable	Minimum	Maximum	Unit
DEM	‒15	5425	Meters
SOC	2.96	136.45	in t C ha-1
HMTS	0	0.99	–
Bio12	59	1355	Milimeters
Bio14	0	46	Milimeters
Temperature	−13.6	27.6	Degrees celsius
Z-Score of mean LST in Summer	−5.22	5.74	–
Z-Score of mean LST in Autumn	−5.09	5.41	–
WEI	0.73	1.32	Index
LULC	1	26	Class

#### Maximum entropy model.

The Maximum Entropy approach (MaxEnt) is commonly used in ML as a non-linear regression approach for predicting wildfires. In this method, past wildfire occurrences are used for predictive wildfire mapping [76,77]. MaxEnt is widely accepted due to its unique characteristics, such as compatibility with high-resolution input data, insensitivity to multicollinearity, and strong forecasting performance [78]. The maximum entropy method has been widely applied in studies assessing ignition potential [76,77,79]. After identifying areas with significant increases in wildfire density, all wildfire points from 2001 to 2023 that fell within these significant trend zones were extracted from the initial dataset (188,766 and 132,986 samples for summer and autumn, respectively). Given the large number of points, duplicate points were first removed using the “Deleting Duplicate Features” function. To further spatially rarefy the occurrence data, points within 1, 3, and 5 kilometers of each other were eliminated. This step has the advantage of reducing statistical bias in correlation-based modeling [65]. Non-correlated points and variables were then analyzed in MaxEnt 3.4.4 [80]. MaxEnt uses two key parameters: the regularization multiplier and feature classes. Finding the optimal values for these parameters before running the model is essential. In this study, the best parameters were determined using the ENMeval 2.0.4 package [81]. Various trials tested regularization multipliers between 1 and 3, with feature classes L, LQ, H, LQH, LQHP, and LQHPT. Each reduced autocorrelation interval involved 10 model replicates, and background points were adjusted to 10,000, 100,000, 150,000, and 200,000, respectively. Modify the following sentence slightly, keeping the original meaning: These points are used to help the model understand the environmental conditions across the entire study area, not just where the species or event is known to occur. These points aid the model in comprehending the environmental conditions throughout the entire study area, rather than solely at locations where the species or event is confirmed to occur Modifying the background points improves model robustness and better represents environmental gradients [82]. Additionally, performance was assessed using the area under the curve (AUC) metric. AUC values of 0.5–0.7 indicate a weak model, 0.7–0.9 a moderate model, and values above 0.9 represent excellent models [83]. Given the large dataset, 70% of the points were used for training and 30% for testing. The jackknife test was applied to assess the importance of variables in different seasons. The Jackknife test in the MaxEnt model helps identify the predictors (factors) that significantly contribute to the probability of fire occurrence distributions, including both current and potential distributions [65]. To compare wildfire probability maps for each season, the difference between the two seasonal maps was calculated using the following formula in ArcMap:


$$abs\left(b-a\right)/max\left(abs\left(b-a\right)\right)$$

In this formula, “a” and “b” represent wildfire risk probability for summer and autumn, respectively [31]. The abs (b - a) calculates the absolute difference in wildfire risk probabilities between the two seasons, highlighting changes in risk. The max (abs (b - a)) is used for normalization, ensuring that the differences are scaled relative to the maximum observed difference. This helps in comparing and interpreting the changes more effectively.

## 3. Results

### 3.1. Fire regime

**Fig 2:**
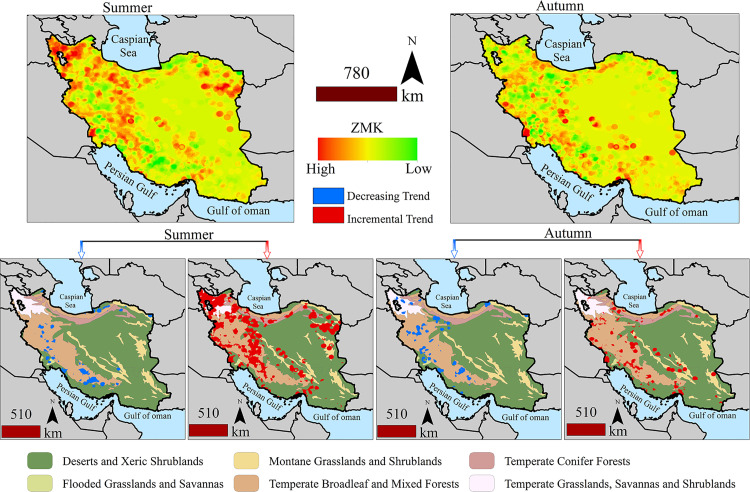
The upper plots show the changes in ZMK for summer and autumn. Red indicates an increasing trend and green indicates a decreasing trend in fire density. The lower plots show significant increasing and decreasing trends for different biomes in Iran.

During the summer season, regions with increasing fire density trends are primarily located in the northwest, northeast, and central parts of Iran. Conversely, in autumn, these areas demonstrating increasing trends are more widely distributed across the country. Fig 2 illustrates the ZMK map, depicting changes in fire density during the summer and autumn seasons. The lower maps within Fig 2 use red and blue colors to denote regions with increasing or decreasing fire density trends, respectively, at the 90% significance level. Notably, the number and size of red patches are more prominent in summer compared to autumn, whereas the blue patches display the opposite pattern, being more extensive and numerous in autumn than in summer.

#### Spatiotemporal changes in the fire regime.

**Fig 3:**
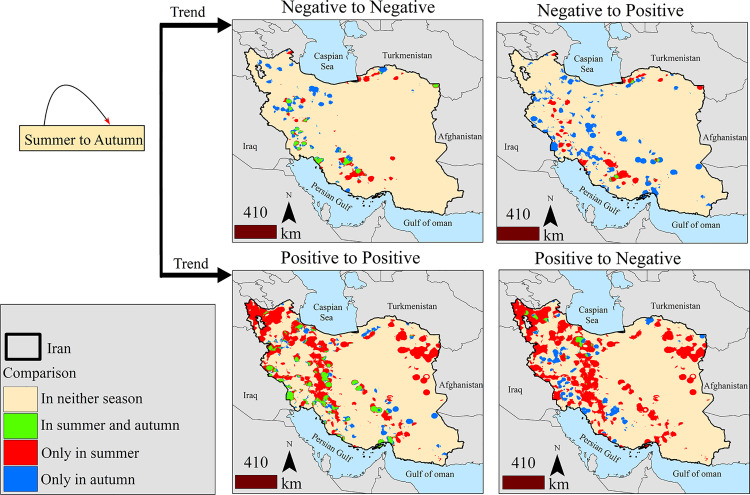
Binary comparison matrix of increasing and decreasing trends in fire density from summer to autumn and comparison of different cases of spatial overlap of these spatial trends.

Significant increases and decreases in fire density showed that the increase in summer covered a larger part of Iran compared to autumn, and the increase in summer and autumn had a high overlap. In autumn, a large part of the fire density shifted to lower latitudes. Fig 3 shows a matrix of changes in densities relative to each other. Different colors in the figure represent various trend classes. Green areas represent regions where both seasons share the same trend, red areas indicate regions where significant trends were only observed in summer, and blue areas indicate regions where significant trends were only observed in autumn. The greatest spatial overlap between summer and autumn trends occurs in areas with decreasing fire density trends. In contrast, increasing trends in summer show the most differences when compared to increasing and decreasing trends in autumn.

Regions with decreasing trends in both summer and autumn cover 18,871.25 km^2^. Areas showing decreasing trends only in summer span 34,415.12 km^2^, while those with decreasing trends only in autumn span 42,213.76 km^2^. A total of 29% of the areas with decreasing trends are spatially aligned. The histogram Kappa index indicates that 0.93 of the regions with increasing and decreasing trends overlap across both seasons, though the location kappa index shows only 0.32 of these regions have spatial alignment. Table 2 compares the area of the classes in the binary maps in Fig 3, highlighting the extent of spatial agreement in summer and autumn.

**Table 2:** Area and overlap between regions exhibiting significant trends in summer and autumn (in km^2^).

	Decreasing in autumn			
Trend	In both	Only summer	Only autumn	Overall, location, and histogram Kappa
Decreasing in summer	18871.25	34415.12	42213.76	0.29; 0.32 and 0.93
	Increasing in autumn			
Trend	In both	Only summer	Only autumn	Overall, location, and histogram Kappa
Decreasing in summer	3329.24	50959.52	99148.61	−0.0; − 0.0 and 0.67
	Increasing in autumn			
Trend	In both	Only summer	Only autumn	Overall, location, and histogram Kappa
Increasing in summer	58134.81	264937.07	44350.12	0.19; 0.45 and 0.41
	Decreasing in autumn			
Trend	In both	Only summer	Only autumn	Overall, location, and histogram Kappa
Increasing in summer	8623.20	314442.19	52443.94	−0.06; − 0.02 and 0.23

#### Influence zones.

**Fig 4:**
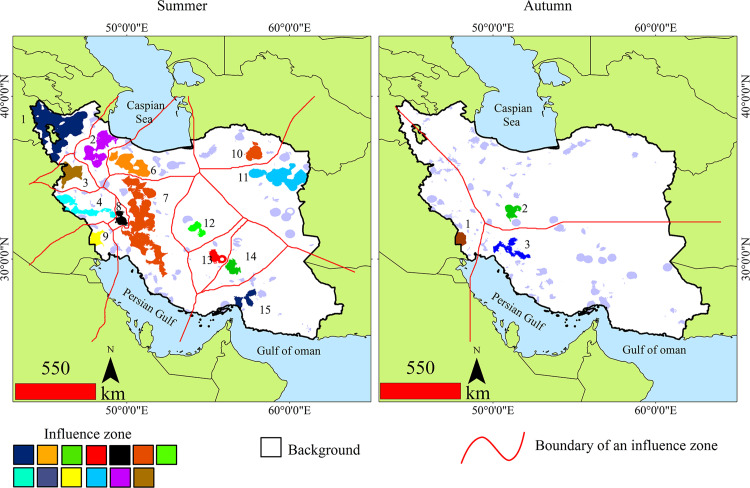
Fire influence zones in Iran, divided by influence area and boundaries between areas, divided by summer and autumn.

Influence Zones will be calculated based on the Euclidean distance map of the background area and the identified Zones will be morphologically separated from other Zones. Fig 4. presents the results of Influence zone analysis across Iran. During summer, Influence zones are scattered across the country. A total of 15 active Influence zones were identified in summer, compared to only 3 in autumn. In summer, the zones are widely dispersed, while in autumn, they are concentrated in the southwestern and central parts of the country. zone 7 (observed in summer) was the largest fire zone. In autumn, zone 3 covered the largest area.

### 3.2. Fire risk

#### Validation and prediction map.

For summer, the best modeling results were achieved using a regularization multiplier of 1 while using 200,000 background points, a 5-km distance, and LQHPT. For autumn, the optimal results were obtained with a regularization multiplier of 1, using 200,000 background points, a 2-km distance, and LQHPT. The AUC scores for the training data were 0.86 for summer and 0.90 for autumn. For the test data, these scores were 0.85 and 0.89, respectively, indicating strong predictive performance. Model validation demonstrated excellent accuracy. According to a binomial test, the models for both seasons differed significantly from random models (P-value <  0.00).

**Fig 5:**
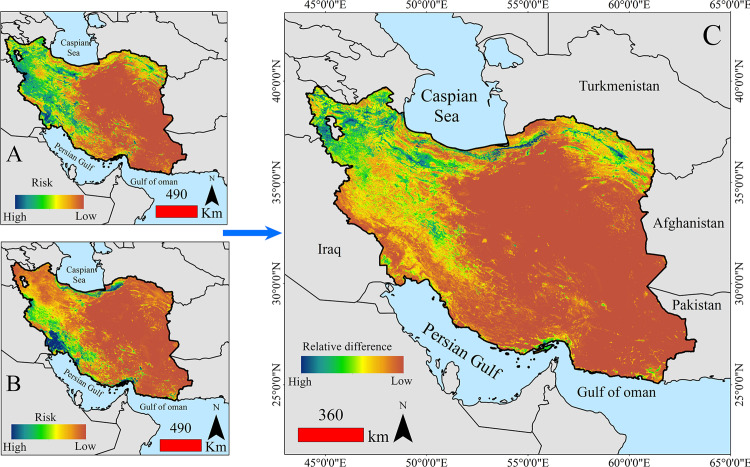
Fire risk prediction maps for summer (A), autumn (B), and the relative differences between the two seasons (C). Blue areas represent regions with a high risk of fire, while brown areas indicate regions with a lower fire risk.

In summer, high-risk areas cover large parts of southern, western, southwestern, northwestern, and northeastern Iran. In autumn, fire risk diminishes in the northwest, but Khuzestan province and the northern strip, particularly Golestan province, show an increased fire risk. Fig 5 presents the results of fire risk prediction and the seasonal variations in fire risk. Fig 5C highlights the major differences between the two seasons, with the most notable variations occurring in the northern strip, northwest, and eastern regions of the country.

#### Influential variables and response curves.

**Fig 6:**
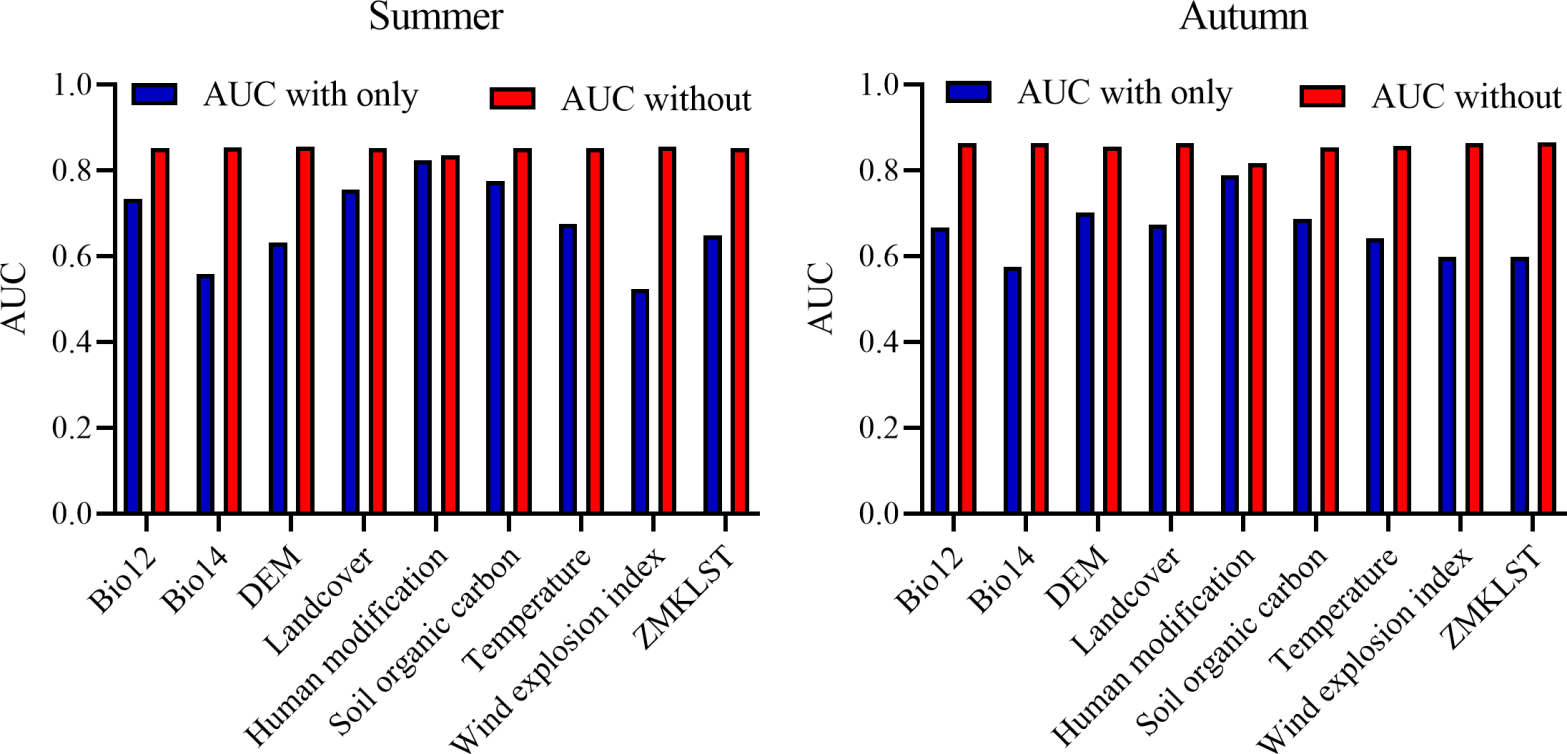
Importance of variables on wildfire occurrence in Iran during summer and autumn based on AUC. Human Modification of Terrestrial Systems (HMTS), Soil Organic Carbon (SOC), and Land Use/Land Cover (LULC) significantly impact fire occurrence in summer, while Human Modification of Terrestrial Systems (HMTS), Soil Organic Carbon (SOC), and Elevation have the most substantial effect in autumn. Wind Exposition Index (WEI) shows the smallest impact on fire occurrence in both seasons.

According to the results, HMTS, SOC, and LULC had the greatest impact on fire occurrence in summer, while HMTS, SOC, and elevation had the most significant impact in autumn. WEI had the smallest effect on fire occurrence in both seasons. Fig 6 presents the importance of variables based on jackknife analysis.

**Fig 7:**
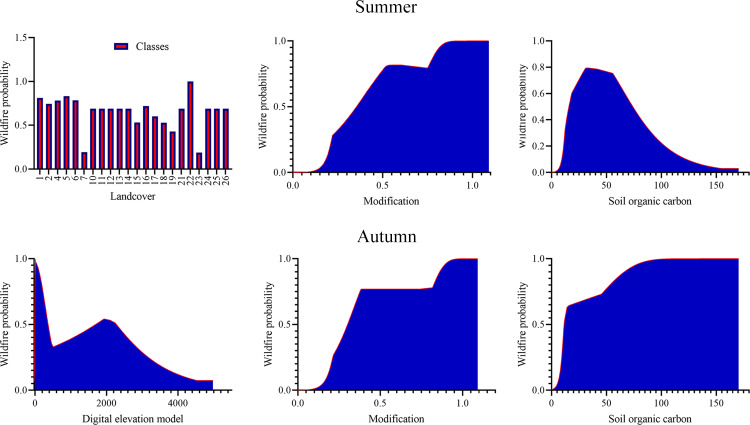
Response curves for key variables influencing wildfire occurrence in summer (top) and autumn (bottom). The graphs illustrate the probability of wildfire occurrence (y-axis) in relation to the values of significant variables (x-axis). The three most impactful variables identified are Human Modification of Terrestrial Systems (HMTS), Soil Organic Carbon (SOC), and Land Use/Land Cover (LULC) for summer, and Human Modification of Terrestrial Systems (HMTS), Soil Organic Carbon (SOC), and Elevation for autumn. Notably, human settlements within the land use categories had the strongest influence on wildfire probability during summer.

A notable difference between the two seasons is the role of land use during summer and elevation in the autumn. In these graphs, the x-axis represents the value of a variable, and the y-axis indicates the probability of wildfire occurrence. In the summer, as SOC increases up to 50, the likelihood of wildfires rises, but beyond that threshold, the probability begins to decline. Among the land use categories, human settlements had the strongest influence on wildfire probability. Fig 7 presents response curves for the three variables with the most significant impact on wildfire occurrence.

## 4. Discussion

### 4.1. Modeling approach

Iran, like many countries in the Middle East, has faced increasingly intense wildfires in recent years. Creating fire history or fire occurrence maps is therefore critical from ecological, social, and economic perspectives [84]. However, no studies have thoroughly examined the spatial distribution trends of fire density across the country. Analyzing historical data on fire density offers an effective tool for creating fire risk maps and developing reliable spatiotemporal models. SRS data with a resolution of 1 km were used to identify fire regimes in Iran, providing valuable insights into large-scale wildfire patterns and dynamics, and allowing for both regional and local analyses [85,86]. This resolution allows for the integration of different datasets from various sources and helps in identifying broad-scale patterns and correlations. While 1 km resolution is useful for capturing general trends and broad patterns in fire probability and spread, finer resolutions (e.g., 30 m or 100 m) offer more detailed and accurate information for localized fire modeling and analysis, which might be crucial for specific areas [28].

In this study, fire density trends from 2001 to 2023 were evaluated using the MK test using MODIS active fire products, identifying regions with increasing fire activity. These areas were then incorporated into the MaxEnt model. Model validation demonstrated excellent accuracy, confirming the effectiveness of the model in predicting fire-prone areas across Iran [87]. In this study, the maximum entropy model showed favorable AUC results for both summer and autumn. A value above 0.8 is generally considered sufficient to ensure the model is generalizable [88]. This integrated methodology enhanced the predictive power of fire risk models, supporting more effective management and mitigation strategies [89]. The study underscores the value of combining statistical trend analysis with ML models to better understand and forecast wildfire occurrences [52]. What is included in this study is the role of time. Time and frequency of occurrence can help identify patterns, and this pattern extraction can lead to predictions of areas where persistent patterns over time may also be observed.

Filtering fire data through trend analysis in SDMs is similar to environmental filtering methods [90]. In environmental filtering, habitats for a species are initially modeled, followed by further modeling based on the predictive map generated by the model [26]. Utilizing this method for fire modeling excludes random fire occurrences, enabling ML algorithms to better detect patterns within the data. Despite the diverse ecological conditions across Iran, the MaxEnt model performed well in identifying high-risk fire areas. The method used in this study demonstrates superior capability in accurately identifying Influence zones compared to traditional ML models and predictive models. Unlike traditional ML models that may rely heavily on predictor variables and training data [91], this method focuses on observed fire trends, leading to more precise and contextually relevant wildfire risk zones.

MK test offers several advantages when analyzing environmental time series, particularly those derived from remote sensing products like MODIS fire data. One of its primary strengths is its nonparametric nature, which obviates the need for the data to conform to a normal distribution—a common requirement in many parametric tests [92,93]. This quality makes it especially robust in handling outliers and skewed data distributions that often occur in ecological and climatic datasets. Additionally, the test is adept at managing missing data without significant loss of power, and it can be adapted to account for seasonal effects, which is crucial when dealing with environmental variables that exhibit periodic behavior [94]. Its reliance on rank-based methods rather than raw data values further enhances its reliability by minimizing the influence of extreme values, thereby providing a more stable assessment of underlying trends in observational records [95]. ZMK in this study emphasized fire density. It is possible that the distance of fire points to each other can also be used as a criterion for outlier detection. Therefore, it is suggested that future studies use this feature.

### 4.2. Trends and influence zones

Seasonal analysis of wildfires reveals distinct patterns in summer and autumn. The areas showing increasing fire trends are more extensive during summer, and the fires are more intense (Fig 4). This rise in fire activity during summer may be due to heightened evaporation of soil moisture, which increases flammable vegetation [96]. Additionally, factors such as rising temperatures, reduced humidity, early snowmelt, and delayed autumn rainfall—all linked to climate change—further contribute to this trend. Several regions experiencing increased fire density in summer are located in the Zagros Mountains, overlapping with the Zagros forests, which are highly vulnerable to summer fires. In recent years, decreased rainfall and rising temperatures have intensified drought conditions across Iran, posing a significant threat to these forests. Drought increases the likelihood of ignition and the rate of fire spread [97]. A study by Jaafari et al. (2019) [98] found that wildfires in the Zagros forests tend to peak in July and August. Similar findings were reported by Beygi Heidarlou et al. (2024) [51] in northern Zagros, consistent with the results of this study in western Iran. Another study by Sayahnia et al. (2024) [12] identified fire clusters in the Zagros region, consistent with the influence zones found in this study. However, our analysis also detected influence zones in the central Zagros.

Fire density trends in the Hyrcanian forests show no significant increase in summer wildfires (Fig 4). Other studies have also identified forest areas with lower wildfire risk. For instance, Ashton and Zhu (2020) [99] found that evergreen, semi-evergreen, and montane temperate forests have the lowest wildfire probability. The moisture retained in vegetation and soil in these forests likely helps suppress fire activity. In dense forests, the tree canopy prevents sunlight from reaching the forest floor, reducing the growth of ground vegetation. Additionally, forest interiors tend to have lower temperatures and higher humidity compared to the outside environment, which helps maintain moisture levels. In contrast, the Zagros forests are sparser, allowing direct sunlight to reach the forest floor, resulting in significantly lower moisture levels compared to the Hyrcanian forests. Heisler et al. (2004) [100] also highlighted that the lack of vegetation cover can lead to higher soil temperatures, further exacerbating fire risk.

Analyzing wildfires on a seasonal basis provides valuable spatiotemporal insights that cannot be fully captured without examining at least two temporal scales. A recent analysis of fire trends has uncovered significant seasonal shifts in fire density between summer and autumn. Certain areas experience an increase in fire density during the summer, followed by a decrease in the autumn. This seasonal variation in wildfire activity can be attributed to factors such as climate fluctuations, changes in vegetation moisture, and human activity. Understanding these patterns is critical for developing targeted wildfire management strategies that address the specific challenges posed by seasonal fire dynamics. Asynchronous wildfire dynamics can significantly affect biodiversity in both agricultural and natural ecosystems within affected regions. These dynamics can also contribute to vegetation degradation in natural habitats. Vegetation degradation reduces the capacity of land to sustain life, impacting wildlife, livestock, agriculture, and humans [59]. Zha et al. (2024) [101] emphasize the importance of managing asynchronous wildfire patterns to ensure accurate predictions. Without understanding these cycles, effective land management is unattainable [102]. For instance, influence zones 7 and 9 in summer correspond to zones 1, 2, and 3 in autumn. A total of 58,134.81 km^2^ exhibit increasing fire trends across both seasons, with these areas experiencing extended fire durations. While fire can enhance habitat heterogeneity, its negative impacts on certain vertebrate species should not be overlooked [31]. Notable species within the Zagros influence zones include the fire salamander (*Salamandra infraimmaculata*), Kurdistan newt (*Neurergus derjugini*), Luristan newt (*Neurergus kaiseri*), wild goat (*Capra aegagrus*), wild sheep (*Ovis orientalis*), roe deer (*Capreolus capreolus*), Persian leopard (*Panthera pardus*), brown bear (*Ursus arctos*), and Persian squirrel (*Sciurus anomalus*) [74].

However, fire duration can serve as a limiting factor. Fire duration is a critical determinant of environmental damage [103] as it affects soil conditions by raising temperatures significantly. When fire heats the topsoil to 250°C, temperatures at a depth of 20 cm can rise to 100°C [104]. Such temperature increases lead to alterations in processes like mineralization, volatilization, and the solubilization of organic compounds at these depths [105]. Vegetation is also affected by fire, with responses varying depending on the ecosystem. Successive fires of varying intensities and durations may select different plant species depending on the fire regime and plant traits [106], promoting the growth of pioneer or invasive species [107,108]. A study by Moradizadeh et al. (2020) [109] on semi-arid oak forests in western Iran found that vegetation structure, composition, and species diversity changed after a fire. Ecosystems with increasing or decreasing fire trends may exhibit varying post-fire responses. Research shows that post-fire recovery is taxon-dependent [110,111], and this can be further influenced by rainfall. In areas where fire duration is long but rainfall is low, post-fire recovery is generally poor. The coppice regeneration characteristic of oak forests makes them particularly fire-sensitive, as the species’ dispersal ability plays a critical role in recovery patterns after wildfires [112]. Future studies should explore how these increasing fire trends affect soil properties and plant community structures.

Northwestern Iran, encompassing the provinces of West Azerbaijan, East Azerbaijan, the Arasbaran forests, and the shores of Lake Urmia, has experienced long-term increases in fire activity. The topographic complexity of the region generates strong winds that elevate the risk of fire spread [113]. Arasbaran forests are affected by different degradation factors, among which fuelwood harvesting and livestock grazing are more important than others [114]. West Azerbaijan features part of the Zagros Mountains, with significant elevation changes and varied landscapes. The strong winds generated by the rugged terrain can rapidly spread fires, impacting the local vegetation, including oak and pistachio forests. During summer, wildfires have been observed in densely cultivated areas across much of Iran, from northern Hormozgan (in the south) to Birjand, Esfarayen, and Quchan (in the northeast). Central Iran is characterized by desert ecosystems and limited fuel, and thus lacks significant influence zones. Influence zones 3, 4, and 8, located in the Zagros Mountains, are particularly prone to fires due to their dry, continuous vegetation in summer, which facilitates fire spread. These zones are primarily separated from each other due to fuel discontinuity. Fuel discontinuity in the landscape can lead to substantial changes in fire spread rates [115]. The high connectivity of central influence zones may be due to the proximity of agricultural lands. Additionally, the use of fire for agricultural waste management contributes to increased fire density and continuity.

An influence zone was observed in the large Hoveyzeh Marsh in southwestern Iran. This marsh spans Iran and Iraq, and both sides experience regular wildfires. Due to rising summer temperatures and persistent drought, large portions of the marsh’s vegetation burn each year, releasing pollutants that drift toward cities located to the east of the marsh. The failure to uphold the water rights associated with the marsh has exacerbated this issue. These findings highlight the impact of prolonged drought, water mismanagement, human activities, and climate change on the increasing vulnerability of the marsh to fire. The findings also underscore the urgent need for effective fire management strategies and international cooperation to mitigate risks and protect this ecologically significant region.

### 4.3. Maximum entropy predication

The impact of environmental variables on fire can vary based on geographical regions [116]. Iran, a country with complex topography, diverse climate zones, variable vegetation, and human activities, can pose challenges for the Maxent model, such as variable interactions, heterogeneous data, and even overfitting. In such circumstances, the choice of spatial resolution for environmental variables can significantly impact the model’s performance. According to the jackknife analysis, HMTS, SOC, and LULC were the most influential factors in the summer. In contrast, during the fall, HMTS, SOC, and elevation had the greatest impact on wildfire occurrence. This indicates that human-related factors are among the most significant contributors to wildfires. Wildfires are often linked to human activities such as agriculture and livestock grazing [14]. Moreover, roads, residential areas, and human infrastructure are major factors in wildfires. Numerous studies have shown that a significant proportion of wildfires occur near roads [6,31,65]. Roads provide access to natural and agricultural lands and serve as key infrastructure. Human settlements have also been identified as critical factors, particularly in the summer when they are the dominant land use influencing fire incidents. The location of most agricultural lands near residential areas intensifies this effect. The combined influence of these factors during summer further emphasizes the role of human activity in wildfires. Additionally, increased human disturbances correlate with a higher likelihood of fires. High population density, alongside concentrated man-made structures and residential areas, further raises the probability of wildfires. A study by Jaafari et al. (2022) [117] on wildfires in western Iran confirmed that land use is the most critical factor in wildfire incidents. Similar findings have been reported by Babu et al. (2023) [118], who investigated the effects of forest fires on the Ghats biodiversity hotspot in India.

Response curves indicated that as SOC levels rise, so does the probability of wildfires. In autumn, there are two peaks in SOC, with the second peak—associated with increased wildfire likelihood—occurring in the Hyrcanian forests. This suggests that SOC levels in the northern strip (Hyrcanian forests) are significantly higher than those in the Zagros forests, resulting in a two-part response curve. In the summer, however, the response curve is not divided because the majority of wildfires occur in areas where SOC remains relatively stable. A study by Fadaei et al. (2022) [119] on the effects of fire on soil characteristics in the Hyrcanian forests found that after a fire, the percentage of sand, the percentage of silt, aggregate stability, soil hydrophobicity, organic carbon, organic matter, total nitrogen, absorbable potassium and phosphorus, electrical conductivity, and pH increased significantly. Salgado et al. (2024) [44] similarly found that soil nitrogen levels decrease as fire recurrence increases. The increasing trend of wildfires could have a significant impact on SOC, as more frequent fires lead to the depletion of the labile carbon reserve [45]. Han et al. (2021) [120] further demonstrated that wildfire disturbances reduce the soil’s ability to sequester carbon. Wildfires also impact soil water repellency (SWR). Immediately following a fire, SWR tends to increase, although this effect is short-lived, as SWR decreases rapidly afterwards [121]. However, studies have shown that SWR continues to increase until the ash produced by the fire is washed into the soil by rainfall [121]. Combined with the reduced rainfall in southern Iran, this could lead to severe environmental challenges such as reduced soil fertility (hindering post-fire vegetation recovery) and compromised soil structure (lower aggregate stability, leading to diminished water and air infiltration) [105]. Given these conditions, land degradation becomes a likely outcome. The loss of SOC in dryland farming and grassland regions underscores the pressing need to enhance farming techniques and adopt more effective pasture management practices [122].

Climate change has been implicated in the occurrence of wildfires. This study helps answer whether areas with increasing wildfire trends also show a rise in LST. The response curve for average summer temperatures indicates that wildfire probability increases as temperatures climb to 10°C, then declines, before rising again above 25°C, and finally decreasing at 35°C. A similar pattern is observed in autumn. These response curves suggest two distinct temperature-related wildfire responses—one in warm areas and another in cold areas. The ZMKLST curve reveals a different dynamic. In summer, areas with ZMK values below -1.96 exhibit a wildfire probability of 1, indicating that fires are likely in regions where LST has been decreasing over the past 23 years. As ZMKLST approaches zero (indicating no trend), wildfire probability decreases, but increases again when ZMKLST rises above zero. This pattern holds for autumn as well. The findings highlight that even regions with decreasing LST face high wildfire risk, whereas many studies have focused solely on areas with rising temperatures as being at risk [123,124]. While temperature increases may act as a driver, other factors—especially human activities such as intentional ignition—must also be considered.

Elevation emerged as the third most significant factor in wildfire occurrence during autumn. The response curves in summer and autumn show similar behavior. Wildfire probability rises with elevation up to 20 meters above sea level, especially in areas such the Persian Gulf coast and Qeshm Island. Beyond this elevation, the probability decreases until 500 meters, representing the country’s lowland regions. However, from 800 meters upwards, the likelihood of wildfire increases again, continuing up to 2500 meters. This suggests that wildfire behavior, similar to temperature, can be categorized into lowland and highland zones. These lowland areas are typically located in Hormozgan province in southern Iran and its islands. Other studies also report higher wildfire risk in lower elevations (600 to 800 meters) [118], likely due to reduced humidity. In both seasons, wildfire probability declines above 2500 meters, where reduced accessibility and lower temperatures serve as limiting factors [125].

Forecasts indicate that Golestan province is exposed to significant wildfire risk in autumn. This region is part of the Hyrcanian forests in northern Iran and is highly sensitive to fires. One factor contributing to wildfire concentration in this season, as noted earlier, is moisture levels. In spring and summer, these forests remain green, providing little fuel for fires. However, in fall, with the arrival of westerly winds at lower latitudes, the probability of wildfires increases. Seasonal differences (Fig 5) demonstrate that the elevated wildfire intensity observed in summer (particularly at higher latitudes), decreases in fall, indicating a direct relationship between latitude and fire severity.

This shift emphasizes the uneven timing of seasonal wildfires, influenced by factors like climate change, vegetation moisture levels, and human activity. The study by Eskandari et al. (2021) [87] supports these conclusions, showing how topography and seasonal factors affect fire risk in Iran. Recognizing the spatial and seasonal differences in wildfire risk is essential for developing effective strategies for wildfire management and prevention. This understanding allows for more effective planning and response to the challenges posed by varying wildfire behavior across seasons.

### 4.4. Study limitations

Fire risk is influenced by dynamic factors such as climate change, human activities, and vegetation changes, which might not be fully captured by static models. Many studies in the field of fire prediction and risk assume that variables are constant over time, while the temporal dynamics of these effective variables can affect fire occurrence[11,65,126]. In this study, only the ZMKLST variable was used to identify the two-season temperature dynamics in the landscape. However, Z-Score analysis can be applied to many other variables such as NDVI and other vegetation indices, as well as numerous SRS spectral indices related to human activities. Incorporating these additional variables in the analysis is suggested to better account for landscape dynamics. By evaluating changes in environmental dynamics alongside fire occurrences, the quality and quantity of landscape components can be more accurately assessed.

Although this study attempted to use the optimal Maxent configuration, the modeling faces inherent shortcomings. Maxent is designed for presence-only data, which means it doesn’t consider absence data [127]. This can lead to biased predictions if the presence data is not representative of the entire study area. Choosing the right environmental variables is crucial, and incorrect or incomplete variable selection can affect model accuracy. Furthermore, the Maxent model can be susceptible to overfitting, especially with complex models or when too many environmental variables are used. Overfitting can lead to models that perform well on training data but poorly on independent test data. Additionally, dynamic human activities and interventions, such as fire suppression efforts and land management practices, are not fully captured in the model, which may lead to potential inaccuracies in fire risk predictions.

The fire data were sourced from the MODIS active fire product, which has been shown to perform poorly in detecting fires smaller than 1 hectare [54]. This means that the satellite active fire data are biased towards detecting larger fires, potentially missing smaller fires within the same pixel as a larger fire. Additionally, the detection algorithm used by MODIS is more likely to identify and report larger, more intense fires due to their higher thermal signature. This limitation affects the reliability of the results. The active fire product detects fires in near real-time, providing immediate information on fire locations and intensities. However, for studying the effects of fire on the environment, the MODIS Burned Area Product might be more suitable, as it provides detailed information on the area affected by fires. This product offers higher spatial resolution, allowing for a more accurate assessment of the extent of burned areas.

## 5. Conclusion

This study introduces an innovative approach to analyzing wildfire regimes by integrating the non-parametric MK test with the Maxent method. This combination offers deeper insights into the spatial patterns of frequent wildfires and high-risk areas, potentially serving as an effective input filter for ML models targeting fire risk. Temporal analysis, incorporating fire density calculations from 2001 to 2023, addresses the dynamic nature of wildfire occurrences, enhancing the understanding of wildfire risk and frequency. The identification of high-risk areas through trend analysis with Maxent modeling enhances the efficiency and accuracy of predictive modeling. Additionally, the application of the ENMeval package to determine optimal Maxent settings ensures robustness and reliability, contributing to the methodological rigor of the study. By applying this approach to a coarse-scale study area encompassing the entire country of Iran, which presents significant topographic and environmental variability, we provide a comprehensive analysis of wildfire patterns across diverse regions. The analysis shows that Iran faces widespread and intense wildfires in summer. In autumn, there are significant wildfire density trends across the country, although the number of Influence zones is notably lower. The fire pattern varies according to the season, and Arasbaran and Zagros forests are at greater risk of fire in summer and Hyrcanian forests in autumn. The greater extent of fire in the summer season can be due to the increase in temperature and the availability of parameters effective on fire in that season. The identified fire zones can be controlled and managed with specific policies for each zone, and the identification of these policies requires knowledge of the existing conditions of each area. For example, for forest areas, a protection policy can be considered, and for pasture areas, continuous monitoring can be considered. Human activities play a clear and effective role in fires in Iran, and this indicates that fire management plans should emphasize these factors. Increasing awareness and informing the role of this component is very necessary and essential for public awareness. Raising public awareness among farmers working in fire-prone areas about better agricultural waste disposal methods is another key preventative measure.

## Supporting information

S1 File https://figshare.com/articles/dataset/_b_Non-parametric_spatiotemporal_trends_in_fire_an_approach_to_identify_fire_regimes_variations_and_predict_seasonal_effects_of_fire_in_Iran_b_/28425032
(ZIP)
